# Ku2: A Novel Korean Purple-Green Tea Germplasm (*Camellia sinensis*) with Enhanced Polyphenols and Antioxidant Activity

**DOI:** 10.3390/plants14172742

**Published:** 2025-09-02

**Authors:** Yun-Suk Kwon, Doo-Gyung Moon, Ha Rim Hong, Byung-Hyuk Kim, Eun Young Song, Chun Hwan Kim, Su Jin Kim

**Affiliations:** Research Institute of Climate Change and Agriculture, National Institute of Horticultural and Herbal Science (NIHHS), Rural Development Administration (RDA), Jeju 63240, Republic of Korea; ys0727@korea.kr (Y.-S.K.); dgmoon@korea.kr (D.-G.M.); gkfla1206@korea.kr (H.R.H.); tiger417@korea.kr (B.-H.K.); eysong@korea.kr (E.Y.S.); kimchw@korea.kr (C.H.K.)

**Keywords:** antioxidant activity, *Camellia sinensis*, colored-leaf tea, epigallocatechin gallate, phenolic compounds

## Abstract

Although colored-leaf tea germplasms can broaden product diversity and functional potential, such resources have been rarely reported in Korea. Herein, we comprehensively characterized Ku2, a newly discovered purple-green line of *Camellia sinensis*, and benchmarked it against the conventional green-leaf ‘Sangmok’. Five-year-old plants grown under identical open-field conditions were evaluated for growth characteristics, leaf pigmentation, biochemical composition, and antioxidant capacity. Ku2 exhibited a more vigorous growth habit with denser branching and produced leaves that were 11% longer and 17% wider than those of ‘Sangmok’, but chlorophyll concentrations were 29–33% lower. Young shoots of Ku2 in the first flush accumulated markedly higher levels of total polyphenols (+38%), anthocyanins (+78%), and total catechins (+35%), including a 70% increase in epigallocatechin-3-gallate. But amino acid and theanine contents were reduced to 30% and 25% of those in ‘Sangmok’, respectively. Consistent with its polyphenol enrichment, Ku2 extracts displayed superior radical-scavenging activity, with lower DPPH and ABTS IC_50_ values (7.6 ± 0.5 and 11.6 ± 0.2 µg·mL^−1^) than ‘Sangmok’ (10.1 ± 0.4 and 15.1 ± 0.1 µg·mL^−1^), approaching ascorbic acid and Trolox standards. These findings highlight Ku2 as a valuable germplasm for developing premium Korean teas and for breeding colored-leaf cultivars enriched with health-promoting metabolites.

## 1. Introduction

Tea is the most-consumed non-alcohol beverage worldwide. Commercial teas are manufactured from young shoots of tea plants (*Camellia sinensis*), typically consisting of one bud with one, two, or three leaves. Depending on the processing method, the degree of polyphenol oxidation by polyphenol oxidase varies, and teas are accordingly classified into non-oxidized (green and yellow teas), minimally oxidized (white tea), semi-oxidized (oolong teas), fully oxidized (black tea), and microbial-fermented (dark tea) types [1].

Tea plants are subtropical crops that belong to the family *Theaceae* and are cultivated and processed into various tea types worldwide [1]. Tea plants originated from the Yunnan-Guizhou Plateau region of China and gradually migrated to other regions and countries. They can be mainly divided into large leaf variety (*C*. *sinensis* var. *assamica*) and medium-small leaf variety (*C*. *sinensis* var. *sinensis*) [2,3,4]. The large-leaf types, which originated in the Assam region of northern India and later spread to other countries, have larger leaves and are more susceptible to cold than the small-leaf types. In contrast, the small-leaf variety originated in China and exhibits greater cold tolerance [5].

Among tea plant varieties, those with green leaves are the most common. However, there are also colored tea plants with purple, purple-red, white, or yellowish- white leaves [6,7]. These non-green-leaf tea plants exhibit distinct nutritional compositions. Albino tea plants with white and/or yellow leaves are rare cultivars, but they contain high levels of amino acids and theanine, both of which are important components determining tea quality [8,9,10]. Tea leaves with purple coloration are characterized by higher level of anthocyanins, which contribute not only to leaf color but also to health-promoting properties. Purple tea cultivars have been identified in China, Japan, Kenya, and India [6,11,12,13]. Among them, ‘Zijuan’ and ‘Ziyan’ are widely cultivated in China, ‘Sunrouge’ in Japan, and ‘TRFK306′ in Kenya [14]. Young purple leaves are processed into various tea types, including green, black, and pu-erh tea, and anthocyanins influence the color, aroma, and taste of the final products [12,15]. Thus, certain compounds in non-green color leaves affect not only the plant’s appearance but also the unique flavor, color, and potential health benefits of tea made from them. For these reasons, consumer interest in colored-leaf teas is growing. Therefore, the development and conservation of diverse colored-leaf tea germplasm is essential for diversifying tea products, meeting market demands, and enhancing the global tea industry.

In Korea, most cultivated and wild-growing tea plants belong to small-leaf species with green foliage [16]. Most tea cultivars registered in the Korea Seed & Variety Service (KSVS, https://www.seed.go.kr/seed/index.do, accessed on 1 September 2025) have green leaves. Among them, *C. sinensis* cv. Sangmok is a nationally registered green-leaf cultivar and is considered a representative Korean landrace tea plant in Korea [17]. In 2023 and 2025, our research team reported on ‘Geumda’, the first yellow-leaf tea cultivar in Korea, which exhibited high levels of free amino acids and theanine [18,19]. Except for ‘Geumda’; however, there have been few reports of colored-leaf tea germplasms in Korea. Recently, our team identified a novel tea germplasm with purple-green leaves. In this study, we describe its growth characteristics and analyze the composition of key biochemical compounds found in this unique Korean purple-green tea line.

## 2. Results

### 2.1. Phenotypic Characterization

**Figure 1:**
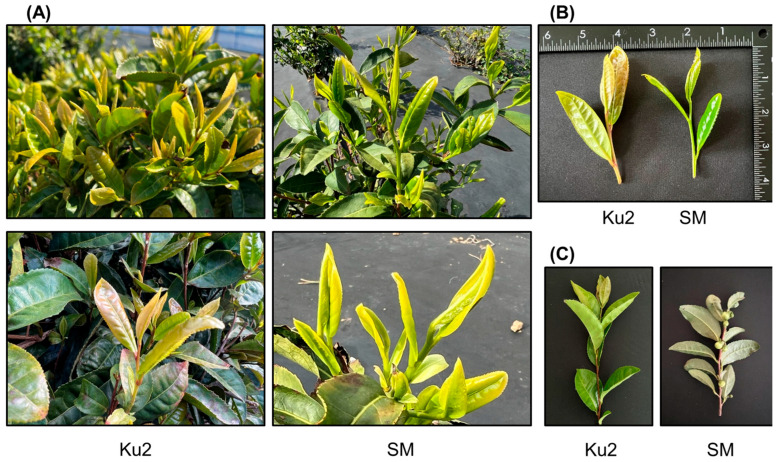
Morphological comparison of Ku2 and *Camellia sinensis* ‘Sangmok’ (SM) at first flush. (**A**) One-bud–three-leaf shoots. (**B**) Third fully expanded leaves (adaxial surface). (**C**) Flowering habit: Ku2 (left) shows no floral buds, whereas SM (right) flowers in early October.

Ku2 is a naturally occurring purple-green tea germplasm identified in Gurye-gun, Jeollanam-do, Republic of Korea, and subsequently propagated and maintained under open-field conditions at Research Institute of Climate Change and Agriculture (RICCA), Jeju, Republic of Korea. Ku2 is not a registered cultivar but rather a breeding line under evaluation as a genetic resource, and it is currently under patent application review (Application No. 10-2025-0123456). For comparison, the green-leaf cultivar ‘Sangmok’ (SM), officially registered by the Rural Development Administration in 2014, was used as the reference cultivar. The detailed origins of Ku2 and SM are described in Section 4.1. During the first harvest, Ku2 exhibited a distinct purple-green coloration in one-bud-three-leaf shoots, while SM displayed a uniform green appearance (Figure 1A,B). As presented in Table 1, Ku2 had a semi-upright growth habit similar to SM but showed more vigorous growth and denser branching. At the ‘two and a bud’ stage, the second leaf was purple-green in Ku2 and yellow-green in SM. Anthocyanin pigmentation was visible at the base of the Ku2 petiole but absent in SM. Shoot length at the ‘three and a bud’ stage was shorter in Ku2.

Ku2 leaves were medium-elliptic, upward-growing, and longer and narrower than those of SM, with lighter green coloration. The cross-section of Ku2 leaves was upwardly folded with moderate rugosity on the upper surface, whereas SM leaves showed flat and smooth. Other traits such as leaf apex and base shape, margin undulation, and serration were comparable between the two lines.

Notably, while SM produced flower buds annually from July to August and bloomed from September to October, no floral bud development or flowering was observed in Ku2 throughout the entire study period (Figure 1C). This absence of flowering was consistent in both the original Ku2 plants and their vegetatively propagated cuttings.

The length, width, and thickness of Ku2 leaves were 9.16 ± 0.71 cm, 3.98 ± 0.22 cm, and 0.38 ± 0.04 mm, respectively (Table 2). In comparison, SM showed values of 8.22 ± 0.48 cm, 3.41 ± 0.31 cm, and 0.47 ± 0.06 mm. Thus, Ku2 leaves were 11–17% larger in length and width, but 19% thinner than those of SM.

### 2.2. Leaf Chlorophyll and Color Indices

**Figure 2:**
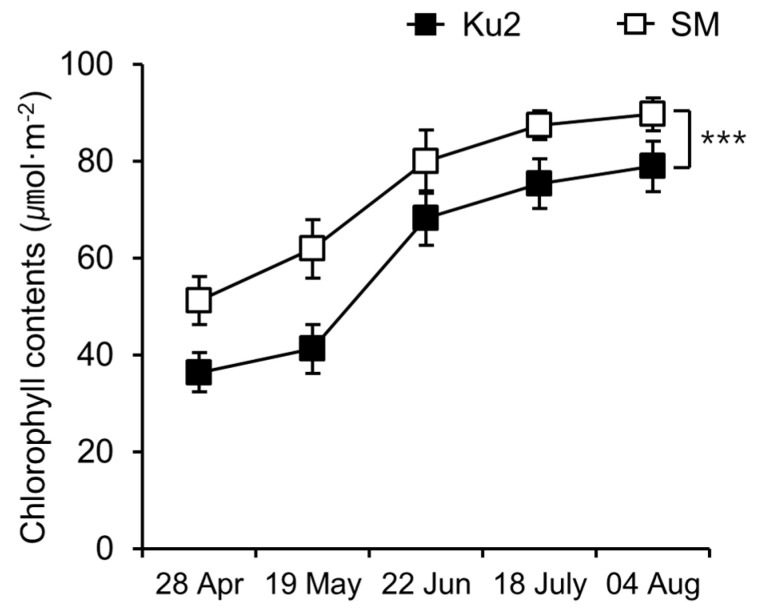
Seasonal changes in chlorophyll content of Ku2 and *Camellia sinensis* ‘Sangmok’ (SM) leaves. Chlorophyll was measured on the second leaf using a SPAD-502 m from April to August 2022. Values are the means ± SD (*n* = 25). Asterisks indicate significant differences between the two lines (*** *p* < 0.001, Student’s *t*-test).

Chlorophyll content of Ku2 at the early (28 April) and the late (19 May) stages of the first flush harvest was 36.4 ± 4.0 µmol·m^−2^ and 41.2 ± 5.1 µmol·m^−2^, respectively (Figure 2), corresponding to 71% and 67% of the levels observed in SM (51.2 ± 4.9 and 61.9 ± 6.0 µmol·m^−2^, respectively). Although chlorophyll content in Ku2 increased steadily until August 4, it remained significantly lower than SM throughout the period.

Color index analysis on the second leaf from the apex during the first flush revealed significant difference between Ku2 and SM (Table 3). Ku showed L*, a*, and b* of 38.46 ± 2.38, −1.27 ± 4.12, and 19.48 ± 3.76, respectively, while SM exhibited 42.35 ± 3.02, −14.07 ± 3.36, and 26.43 ± 4.34. These results indicate that Ku2 leaves were darker (lower L*), less green (higher a*), and less yellow (lower b*) than those of SM.

### 2.3. Primary Non-Volatile Metabolites

**Figure 3:**
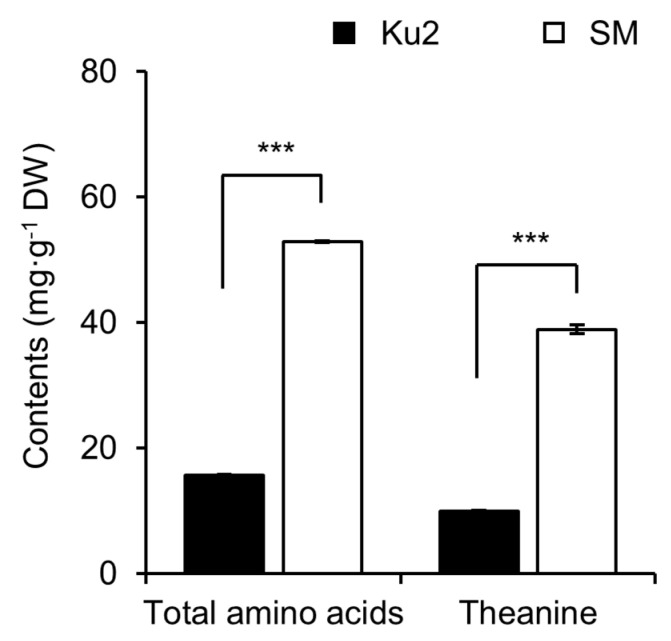
Total amino acids and theanine concentrations in young shoots of Ku2 and *Camellia sinensis* ‘Sangmok’ (SM). One-bud-three-leaf samples were collected at first flush. Values are the means ± SD (*n* = 3). Asterisks indicate significant differences between the two lines (*** *p* < 0.001, Student’s *t*-test).

The total amino acid content was 15.71 ± 0.15 mg·g^−1^ DW in Ku2 and 52.90 ± 0.16 mg·g^−1^ DW in SM, while theanine content was 9.93 ± 0.15 mg·g^−1^ DW in Ku2 and 38.91 ± 0.73 mg·g^−1^ DW in SM (Figure 3). These results indicate that Ku2 had approximately 30% and 26% of the total amino acid and theanine contents in SM.

**Figure 4:**
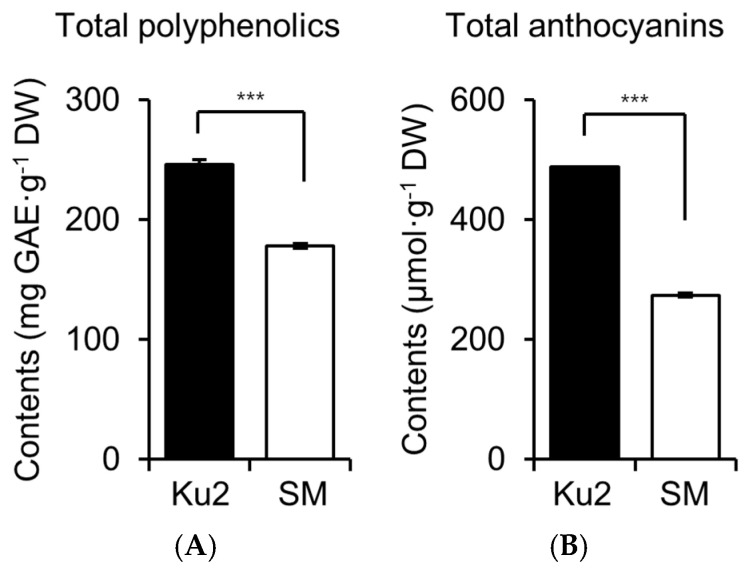
Total polyphenol (**A**) and anthocyanin (**B**) contents in young shoots of Ku2 and *Camellia sinensis* ‘Sangmok’ (SM). Polyphenols are expressed as gallic acid equivalents (GAE); anthocyanins as µmol·g^−1^ DW. Values are the means ± SD (*n* = 3). Asterisks indicate significant differences between the two lines (*** *p* < 0.001, Student’s *t*-test).

In contrast, Ku2 accumulated higher total polyphenol and anthocyanin contents than SM (Figure 4A). Total polyphenols were 245.8 ± 4.4 mg GAE·g^−1^ DW in Ku2 and 178.0 ± 1.7 GAE mg·g^−1^ DW in SM, indicating that Ku2 contained approximately 1.4-fold higher content. Total anthocyanin content was 487.7 ± 0.6 µmol·g^−1^ DW in Ku2 and 273.7 ± 3.4 µmol·g^−1^ DW in SM, reflecting a 1.8-fold increase in Ku2 (Figure 4B).

**Figure 5:**
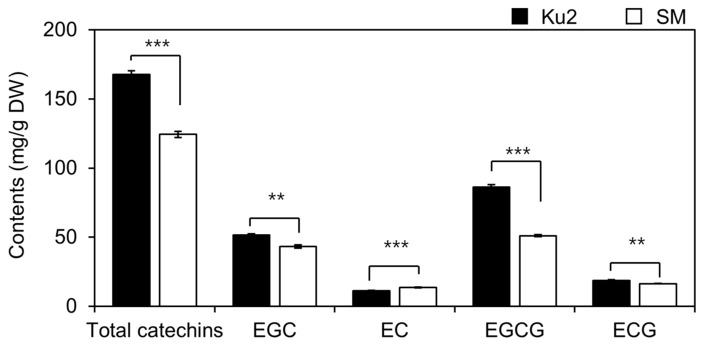
Catechin profile of Ku2 and *Camellia sinensis* ‘Sangmok’ (SM) leaves at first flush. EGCG, ECG, EGC, EC, and total catechins are given in mg·g^−1^ DW. Values are the means ± SD (*n* = 3). Asterisks (*) indicate significant differences between the two lines, as determined by Student’s *t*-test (** *p* < 0.01; *** *p* < 0.001).

Regarding catechins, Ku2 showed higher levels of EGCG (86.1 ± 1.9 mg·g^−1^ DW), EGC (51.6 ± 0.8 mg·g^−1^ DW), and ECG (18.7 ± 0.4 mg·g^−1^ DW) than SM (51.1 ± 0.8, 43.3 ± 1.3, and 16.4 ± 0.2 mg·g^−1^ DW, respectively), corresponding to 1.7-, 1.2-, and 1.1-fold increases (Figure 5). Among these, EGCG showed the greatest difference. Only EC was lower in Ku2. The four major catechins (EGCG, ECG, EGC, EC) summed to 167.8 ± 2.7 mg·g^−1^ DW in Ku2 and 124.3 ± 2.3 mg·g^−1^ DW in SM, indicating 1.4-fold increase in Ku2.

### 2.4. Antioxidant Capacity

**Figure 6:**
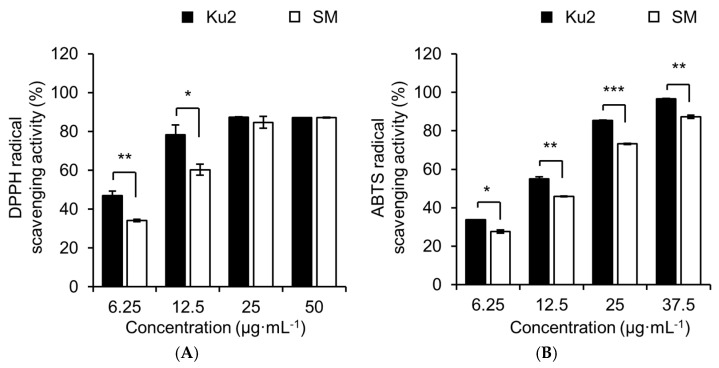
Radical-scavenging activity of 70% ethanol extracts from Ku2 and *Camellia sinensis* ‘Sangmok’ (SM). (**A**) DPPH assay; (**B**) ABTS assay. Ascorbic acid (DPPH) and Trolox (ABTS) served as positive controls. Values are the means ± SD (*n* = 3). IC_50_ values are provided in the text. Asterisks (*) indicate significant differences between the two lines, as determined by Student’s *t*-test (* *p* < 0.05, ** *p* < 0.01, *** *p* < 0.001).

Seventy-percent ethanol extracts of Ku2 exhibited stronger radical-scavenging activity than those of SM in both antioxidant assays (Figure 6). In DPPH assay, Ku2 demonstrated significantly higher radical-scavenging activity, especially low concentrations (Figure 6A). At 6.25 µg·mL^−1^, Ku2 scavenged 46.9 ± 2.4% of DPPH radicals, whereas SM scavenged 34.2 ± 0.6%. At 12.5 µg·mL^−1^, scavenging activity increased to 78.3 ± 5.0% in Ku2 and 60.3 ± 2.8% in SM. The IC_50_ values, representing the concentration required to scavenge 50% of radicals, were 7.6 ± 0.5 µg·mL^−1^ for Ku2, 10.1 ± 0.4 µg·mL^−1^ for SM, and 8.0 ± 0.1 µg·mL^−1^ for ascorbic acid (Table S1).

In the ABTS assay, Ku2 also exhibited stronger activity across all tested concentrations (Figure 6B). The IC_50_ values were 11.6 ± 0.2 µg·mL^−1^ for Ku2, 15.1 ± 0.1 µg·mL^−1^ for SM, and 9.9 ± 0.0 µg·mL^−1^ for Trolox, a vitamin E derivate with strong antioxidant activity [20] (Table S1).

## 3. Discussion

This study characterized the phenotypic and biochemical traits of Ku2, a unique Korean purple-green tea germplasm. Ku2 exhibited a desirable combination of traits, including vigorous growth, visually distinctive foliage, elevated levels of catechin and anthocyanin, and strong antioxidant capacity (Table 1; Figure 1, Figure 4, Figure 5 and Figure 6). Notably, Ku2 had longer and broader leaves, denser branching (Table 1 and Table 2; Figure 1B), and a 1.3-fold greater fresh weight of harvested shoots compared to SM, with the fresh weight of ten Ku2 shoot (one bud and three leaves) being 8.38 g versus 6.39 g for SM. These traits suggest Ku2′s superior leaf biomass productivity, which is advantageous for green tea production, where tender leaf yield is critical. Its enhanced shoot vigor and expanded leaf area may also contribute to improved photosynthetic capacity and adaptability under field conditions, distinguishing it from conventional green-leaf cultivars, such as SM. Together, these attributes imply that Ku2 may harbor unique genetic factors, warranting further investigation of its origin and genetic composition.

Ku2 originates from a native Korean tea population propagated from seedlings on mountain slopes in Gurye-gun, Jeollanam-do, and has been shaped by long-term natural selection. Given the self-incompatibility of tea plants, which promotes genetic diversification, Ku2 likely harbors unique genetic traits. Previous research by Shim et al. [21] demonstrated that ancient Korean teas ‘Hadong Cheon-Nyeon Cha’ show low genetic homology with Chinese and Japanese cultivars, supporting the existence of independently evolved Korean lineages. Based on this, Ku2 may represents a genetically distinct resource with high potential value for breeding and conservation of native tea germplasm in Korea.

Interestingly, Ku2 also exhibits a rare developmental trait: complete absence of floral bud formation over multiple years of cultivation, both in original and vegetatively propagated plants (Figure 1C). This is unusual, given that *Camellia sinensis* is a flowering evergreen species. Floral development in tea is regulated by both environmental factors such as temperature and nutrient status and endogenous regulatory mechanisms [22,23]. This consistent non-flowering trait suggests a deviation from typical developmental regulation and merits further research into its physiological and genetic basis.

Alongside these reproductive traits, Ku2 displays a remarkable biochemical profiles. It accumulated significantly higher levels of total polyphenol (TP), anthocyanins (TA), and four major catechins (EGCG, EGC, EC, and ECG) during the first harvest compared to SM (Figure 4, Figure 5 and Figure 6). Tea polyphenols, including gallic acid, catechins, flavonoids, tannins, and anthocyanins, account for approximately 15–35% of the dry weight of tea leaves [24]. Among these, catechins, especially EGCG, ECG, EC, and EGC, constitute the largest proportion and are primarily responsible for the bitterness and astringency of tea. Gallated catechins (EGCG and ECG) are known to be less bitterness and astringency than non-gallated ones (EGC and EC) [25]. Although tea polyphenols do not directly impart pleasant flavor, they are key determinants of quality and bioactivity of oxidized teas. During oxidation, tea polyphenols are enzymatically converted into theaflavins, thearubigins, and aromatic compounds that define the flavor and color of teas like black and oolong tea [1,26,27]. Catechins also serve as major bioactive compounds linked to antioxidant, anticancer, anti-obesity, anti-inflammatory, hypolipidemic, and antiviral effects [28,29,30], making them a key target in breeding programs. Thus, polyphenol-rich germplasm such as Ku2 are valuable for both quality improvement and functional tea development.

While purple-leaf tea cultivars open contain higher anthocyanins, elevated polyphenol or catechin levels are not always observed [6,11,12,13,31,32]. For example, Ding et al. [6] and Li et al. [31] reported no significant difference in total polyphenol or catechin levels between purplish and green tea leaves. Similarly, Wang et al. [11] and Joshi et al. [12] showed that catechin contents did not increase despite higher polyphenol levels. In contrast, our findings demonstrate that Ku2 is a purple-green-leaf tea germplasm with simultaneous accumulation of high polyphenol, anthocyanins, and catechin levels, especially EGCG.

This distinct metabolic profile may reflect an inverse relationship between amino acid and polyphenol biosynthesis, as previously reported in tea plants [6]. Amino acids influence tea flavor, especially umami and sweetness, and aromas, with theanine accounting for approximately 60–70% of the total free amino acids. Theanine is synthesized from glutamic acid and ethylamine, which in turn is derived from alanine [33]. Ding et al. [6] suggested that ethylamine’s involvement in both amino acid and polyphenol pathway may suppress one biosynthesis pathway when the other is upregulated. Additionally, phenylalanine is converted to coumaroyl-CoA through several enzymatic steps in phenylpropanoid pathway. This intermediate then enters the flavonoid biosynthesis pathway, leading to the production of non-gallated catechins, which serve as precursors to gallated catechins [4,25]. This inverse relationship was also reflected in Ku2, which exhibited high polyphenol accumulation but reduced levels of free amino acids and theanine, at only 30% and 25% of SM levels, respectively (Figure 3, Figure 4 and Figure 5). Given a central role of theanine in contributing to umami flavor, its reduction may impact the flavor profile of Ku2-based teas.

Nonetheless, in addition to the amino acid/catechin ratio, tea flavor quality can also be assessed through the balance of the catechin types [7,29,31,34]. The catechin quality index, calculated as formula [(EGCG + ECG)/EGC], is also used to evaluate green tea quality [7,31]. This index reflects the balance between catechins with relatively mild (EGCG and ECG) and strong (EGC) bitterness and astringency, with higher values indicating better sensory quality. Based on this calculation, Ku2 showed a significantly higher index (2.0 ± 0.0) than in SM (1.6 ± 0.0) (*p* < 0.001), meaning that its overall sensory appeal can be maintained despite lower theanine content. These results suggest that Ku2 represents a promising genetic resource for the development of high-quality tea. Its potential should be further validated through integrated metabolite profiling and sensory evaluations of processed teas derived from Ku2.

In addition to taste and composition, antioxidant activity serves as a key indicator of tea’s functional quality. Polyphenol content is positively correlated with various health-promoting effects, particularly antioxidant activity [35]. In both DPPH and ABTS assays, Ku2 extracts exhibited stronger radical-scavenging activity than SM, with IC50 values comparable to those of ascorbic acid and Trolox (Figure 6 and Supplementary Table S1). Although Ku2 and SM extracts appeared to show similar activities at higher concentrations, this apparent similarity is most likely due to the saturation of radical scavenging at such levels. At lower concentrations (6.25–12.5 µg·mL^−1^), Ku2 consistently exhibited significantly stronger activity than SM, and its IC_50_ values were markedly lower in both DPPH (7.6 ± 0.5 µg·mL^−1^ for Ku2 vs. 10.1 ± 0.4 µg·mL^−1^ for SM) and ABTS assays (11.6 ± 0.2 µg·mL^−1^ for Ku2 vs. 15.1 ± 0.1 µg·mL^−1^ for SM). These results suggest that Ku2 has inherently higher antioxidant capacity, but the differences become less distinguishable at higher concentrations where radical-scavenging approaches maximal levels.

Moreover, the DPPH and ABTS assays differ in their radical forms and solubility characteristics, which influence their interaction with antioxidant groups in tea. DPPH is a stable nitrogen radical soluble in organic solvents and mainly interacts with lipophilic antioxidants such as gallated catechins (e.g., EGCG and ECG), whereas ABTS is soluble in both aqueous and organic media, allowing reactions with a broader spectrum of compounds, including hydrophilic antioxidants such as anthocyanins and flavonols [36,37]. Therefore, the slightly different IC_50_ values observed between DPPH and ABTS assays in Ku2 extracts may reflect the combined contributions of both catechins and anthocyanins. Taken together, these complementary results indicate that the enhanced antioxidant capacity of Ku2 is attributable to its elevated levels of multiple polyphenol groups with distinct radical-scavenging mechanisms. Given these characteristics, Ku2 holds promise for the development of functional tea products that meet the growing consumer demand for bioactive-rich and visually distinctive teas.

In parallel, the combination of pigmented shoots, abundant polyphenols, and strong antioxidant capacity observed in Ku2 highlights its potential as a germplasm resource for breeding novel tea cultivars. In Korea, where breeding of colored-leaf tea cultivars is still in early stages, Ku2 represents a valuable native resource. With Ku2 characterized as a Korean colored-leaf tea germplasm, we believed that it will serve as a foundation for broader genetic and conservation research.

## 4. Materials and Methods

### 4.1. Plant Material

A purple-green-leaf tea germplasm, Ku2, was discovered as a naturally occurring variant in Gurye-gun, Jeollanam-do, Republic of Korea (35°14′9.3″ N, 127°29′9.0″ E). Cuttings taken from the original bushes were rooted at the nursery of Research Institute of Climate Change and Agriculture (RICCA) in Jeju, Republic of Korea (33°28′09.5″ N, 126°30′58.3″ E). Vigorous, morphologically uniform ramets were transplanted to an open-field experimental plot at RICCA, and their first-flush shoots consistently exhibited the same purple-green phenotype. Five-year-old Ku2 plants are used for all observations. The green-leaf tea ‘Sangmok’ (SM), registered by the Rural Development Administration with the KSVS in 2014 (Application No. 2011-426), was used as the control. All cultural practices—propagation, planting density, fertilization, and pest management—were kept identical for Ku2 and SM.

### 4.2. Growth Characteristics and Leaf Size Measurement

The growth characteristics of Ku2 and SM were investigated according to the UPOV examination standards, translated from UPOV (2 September 2020 ver.), provided by KSVS. A total 22 items, consisting of 15 quantitative characteristics, 4 pseudo-qualitative characteristics, and 3 qualitative characteristics, were visually observed and scored.

Leaf dimensions were measured on 8 September 2022 using the third fully expanded leaf from the apex. Length, width, and thickness were recorded with a digital caliper on five plants per line (two leaves per plant). Leaf area was calculated as: Leaf area (cm^2^) = length × width × 0.70.

### 4.3. Determination of Chlorophylls Content and Color Index of Leaves

Chlorophyll content was measured monthly from April to August 2022 on the second leaf from the apex with SPAD-502 m (Konica Minolta, Osaka, Japan). Leaf color (L*, a*, b*) was measured on the same leaf position using a CM700d spectro-colorimeter (Konica Minolta). Five plants per line were tested; each value is the mean of five measurements taken on different leaves per plants.

### 4.4. Quantitative Analysis of Leaf Components

#### 4.4.1. Sample Preparation

During the first flush, young shoots including a bud and three leaves were harvested from five plants per line. The shoots were freeze-dried and ground into a fine powder using a grinder. The leaf powder sample were stored at −20 °C and used for components analysis.

#### 4.4.2. Amino Acids

The leaf powder (0.5 g) was extracted with 10 mL distilled water at 95 °C for 20 min and centrifuged (6000 rpm, 10 min). The supernatant was mixed 1: 1 (*v*/*v*) with 5% (*w*/*v*) trichloroacetic acid, vortexed, and centrifuged (10,000 rpm, 10 min) to separate the protein-free supernatant. This supernatant was diluted tenfold with 0.02 N HCl, filtered through a 0.2 µm nylon syringe filter, and used for amino acid analysis using a high-speed amino acid analyzer (LA8080, Hitachi High-Tech Science Corp., Tokyo, Japan). The analytical conditions were as follows: column-Hitachi custom ion exchange resin (4.6 mm ID × 60 mm); mobile phase-protein hydrolysate analysis buffers (PH Kanto, PH-1, PH-2, PH-3, PH-4, and PJ-RG, Kanto Chemical Co., Inc., Tokyo, Japan); color reagent-Ninhydrin Coloring Solution kit (Fujifilm Wako Pure Chemical Corp., Osaka, Japan); flow rate-0.35 mL·min^−1^; detection wavelengths-570 nm for most amino acids and 440 nm specifically for theanine. Amino acids and theanine content in the freeze-dried tea leaf powder were expressed as mg per g of dry weight (mg·g^−1^ DW). Standard mixtures of amino acids and theanine were obtained from FujiFILM Wako Pure Chemical Corporation.

#### 4.4.3. Total Polyphenols

The leaf powder (0.2 g) was extracted with 70% ethanol (*v*/*v*, 5 mL) under ultrasonication for 30 min. The extract was then centrifuged (13,000 rpm, 10 min), and the supernatant was filtered using a 0.2 µm nylon syringe filter. An aliquot (20 µL) of the 70% ethanol extract solution was mixed with 0.2 M Folin-Ciocalteu reagent (100 µL, Sigma-Aldrich, St. Louis, MO, USA) and incubated for 10 min, followed by the addition of 20% Na_2_CO_3_ solution (*w*/*v*, 30 µL) The mixture was then incubated at 30 °C for 60 min. Absorbance was measured at 765 nm. Gallic acid (Sigma-Aldrich) was used as a standard, and results were expressed as mg of gallic acid equivalents per g of dry weight (mg GAE·g^−1^ DW).

#### 4.4.4. Total Anthocyanins

Total anthocyanin assay was measured following a previously reported method [32]. Leaf powder (0.1 g) was mixed with 0.1 M HCl (10 mL) and incubated at 60 °C for 30 min, shaking every 10 min using a vortex mixer. After centrifugation (13,000 rpm, 5 min), the supernatant was filtered through a 0.2 µm nylon syringe filter. Absorbance was measured at 530, 620, and 650 nm. Anthocyanin content was calculated using the formula: ∆A = (A530 − A620) − 0.1 × (A650 − A620); Total anthocyanin (µmol·g^−1^ DW) = (∆A × 100)/(4.62 × sample weight).

#### 4.4.5. Catechins

The leaf powder (0.02 g) was mixed with methanol (1 mL) using a vortex mixer, sonicated for 25 min, centrifuged (13,000 rpm, 10 min), and the supernatant was filtered using a 0.2 µm nylon syringe filter. Catechin content was analyzed using ultra-performance liquid chromatography (UPLC, Waters Acquity™, Milfold, MA, USA) with an ACQUITY UPLC^®^ HSS T3 column (2.1 × 100 mm, 1.8 µm, Waters). The mobile phase consisted of 0.1% acetic acid in distilled water (A) and 0.1% acetic acid in acetonitrile (B). Gradient elution was performed as follows: 0–3 min, 95% A; 3–12 min, 95–75% A; 12–13 min, 75–40% A; 13–16 min, 40% A; 16–17 min, 40–95% A; 17–22 min, 95% A. Flow rate: 0.2 mL·min^−1^; detection: 280 nm. Catechin content in freeze-dried tea leaf powder was expressed as mg per g of dry weight (mg·g^−1^ DW). Standard compounds (EGCG, ECG, EGC, EC) were purchased from Sigma-Aldrich.

### 4.5. Measurement of Antioxidant Capacity

#### 4.5.1. Preparation of 70% Ethanol Extract

The leaf powder was soaked in 70% ethanol (1:10, *v*/*v*) at room temperature for 24 h. The extract was filtered to remove solids, and extraction was repeated twice using fresh 70% ethanol (1:10, *w*/*v*). All extracts were combined, concentrated in vacuo at 50 °C using a rotary evaporator (Rotavapor R-300, BÜCHI, Flawil, Switzerland), and freeze-dried. The resulting extracts were dissolved in DMSO and stored at −70 °C until use.

#### 4.5.2. Determination of 2,2-Diphenyl-1-picrylhydrazyl (DPPH) Radical-Scavenging Activity

DPPH radical-scavenging activity was analyzed using a modified method based on Brand-Williams et al. [38]. Seventy percent ethanol extracts (20 µL) at various concentrations were mixed with 0.1 mM DPPH in methanol (180 µL). The mixture was incubated in the dark at room temperature for 30 min. Absorbance was measured at 517 nm. DMSO and ascorbic acid (Sigma-Aldrich) were used as negative and positive controls, respectively. DPPH scavenging activity (%) = (1 − Sample absorbance/Control absorbance) × 100.

#### 4.5.3. Determination of 2,2′-Azino-bis(3-ethylbenzothiazoline-6-sulfonic acid) (ABTS) Radical-Scavenging Activity

ABTS radical-scavenging activity was measured using a modified method based on Re et al. [39]. ABTS radicals were generated by mixing 7.4 mM ABTS with 2.6 mM potassium persulfate and incubating the mixture in the dark at room temperature for at least 16 h. The ABTS solution was diluted with distilled water to achieve an absorbance of 1.2–1.3 at 734 nm. For the assay, 70% ethanol extract (5 µL) at various concentrations was incubated with ABTS solution (195 µL) in the dark for 4 min at room temperature, and the absorbance was measured at 734 nm. Trolox (Sigma-Aldrich) was used as a positive control. ABTS scavenging activity (%) = (1 − Sample absorbance/Control absorbance) × 100.

### 4.6. Statistical Analysis

All data are expressed as the mean ± standard deviation (SD) from three independent biological replicates (*n* = 3) per line, each replicate consisted of pooled material from five randomly selected plants. Statistical significance was determined using Student’s *t*-test, with *p* < 0.05 considered significant.

## 5. Conclusions

Ku2 is the one of the few colored-leaf tea lines documented in Korea. It has four key strengths: (i) vigorous growth with enlarged foliage, (ii) a visually striking purple-green shoot, (iii) markedly higher levels of polyphenols, anthocyanins, and catechins, especially EGCG, and (iv) antioxidant activity comparable to that of known reference compounds. Although Ku2 contains less theanine, which may slightly reduce the umami flavor, its high catechin-quality index indicates that bitterness and astringency are still within an acceptable sensory range. Overall, Ku2 offers two major benefits: it expands the variety of Korean tea products and gives breeders a useful plant resource to develop new teas with colored foliage and health-promoting metabolites. Moving forward, sensory evaluations should be conducted to assess consumer acceptance of Ku2-based teas. Field trials at multiple locations are also needed to confirm its yield stability in different environments. In addition, molecular studies should explore the genetic reasons behind its consistent non-flowering trait to support marker-assisted breeding.

## Figures and Tables

**Table 1:** Growth characteristics of the purple-green leaves germplasm (Ku2) and *Camellia sinensis* ‘Sangmok’ (SM).

	Characteristics	Phenotypes	Score	Ku2	SM
1 (*) ^1^ QN ^2^	Plant: vigor	Weak	3	7	5
		Middle	5		
		Strong	7		
2 (*) PQ ^3^	Plant: type	Shrub	1	1	1
		Semi-arbor	2		
		Arbor	3		
3 (*) QN	Plant: growth habit	Upright	1	3	3
		Semi-upright	3		
		Spreading	5		
4 QN	Plant: density of branches	Sparse	3	7	3
		Medium	5		
		Dense	7		
5 QL ^4^	Branch: zigzagging	Absent	1	1	1
		Present	9		
6 PQ	Young shoot: color of second leaf at ‘two and a bud’ stage	White	1	6	4
		Yellow	2		
		Yellow-green	3		
		Light green	4		
		Middle green	5		
		Purple-green	6		
7 (*) QL	Young shoot: pubescence of a bud	Absent	1	9	9
		Present	9		
8 QN	Young shoot: density of pubescence of a bud	Sparse	3	3	3
		Medium	5		
		Dense	7		
9 QL	Young shoot: anthocyanin coloration at base of the petioles	Absent	1	2	1
		Present	9		
10 (*) QN	Young shoot: length of ‘three and a bud’	Short	3	3	5
		Medium	5		
		Long	7		
11 (*) QN	Leaf blade: attitude	Upwards	1	1	3
		Outwards	3		
		Downwards	5		
12 (*) QN	Leaf blade: length	Short	3	7	5
		Medium	5		
		Long	7		
13 (*) QN	Leaf blade: width	Narrow	3	3	5
		Medium	5		
		Broad	7		
14 QN	Leaf blade: shape	Very narrow elliptic	1	3	3
		Narrow elliptic	2		
		Medium elliptic	3		
		Wide elliptic	4		
15 QN	Leaf blade: intensity of green color	Light	3	3	7
		Medium	5		
		Dark	7		
16 QN	Leaf blade: shape in cross-section	Folded upwards	1	1	2
		Flat	2		
		Recurved	3		
17 QN	Leaf blade: texture of upper surface	Smooth or weakly rugose Moderately rugose Strongly rugose	3 5 7	5	3
18 PQ	Leaf blade: shape of apex	Obtuse	1	2	2
		Acute	2		
		Acuminate	3		
19 QN	Leaf blade: undulation of margin	Absent or weak	3	3	3
		Medium	5		
		Strong	7		
20 QN	Leaf blade: serrations on the margin	Weak	3	5	5
		Medium	5		
		Strong	7		
21 PQ	Leaf blade: shape of base	Acute	1	1	1
		Obtuse	2		
		Truncate	3		
22 QN	Time of full flowering	Early	3	No flowers	3 (19 September 2022)
		Medium	5		
		Late	7		

^1^ (*): Important characteristics for the international harmonization of variety descriptions. ^2^ QN: 2uantitative characteristic. ^3^ PQ: pseudo-qualitative characteristic. ^4^ QL: qualitative characteristic.

**Table 2:** Leaf dimension of Ku2 and *Camellia sinensis* ‘Sangmok’ (SM) on 8 September 2022.

Division	Ku2	SM
Length (cm)	9.16 ± 0.71 ***	8.22 ± 0.48
Width (cm)	3.98 ± 0.22 ***	3.41 ± 0.31
Thickness (mm)	0.38 ± 0.04 ***	0.47 ± 0.06

The results are presented as the means ± SD (*n* = 10). Significant differences between Ku2 and SM were determined using Student’s *t*-test, expressed with asterisks (*** *p* < 0.001).

**Table 3:** Colorimeter indices (L*, a*, b*) of the second leaf at first flush in Ku2 and *Camellia sinensis* ‘Sangmok’ (SM).

Parameters	Ku2	SM
L*	38.46 ± 2.38 ***	42.35 ± 3.02
a*	–1.27 ± 4.12 ***	–14.07 ± 3.36
b*	19.48 ± 3.76 ***	26.43 ± 4.34

The results are presented as the means ± SD (*n* = 25). L*: lightness (0 = black, 100 = white), a*: green (–) to red (+); b*: blue (–) to yellow (+). Significant difference between Ku2 and SM was measured by Student’s *t*-test (*** *p* < 0.001).

## Data Availability

The raw data supporting the conclusions of this article will be made. available by the authors upon request.

## Supplementary Materials

The following supporting information can be downloaded at: https://www.mdpi.com/article/10.3390/plants14172742/s1, Table S1: IC_50_ values (µg·mL^−1^) for radical-scavenging activity of 70% ethanol extracts from Ku2 and ‘Sangmok’ (SM).

## Abbreviations

The following abbreviations are used in this manuscript:

DW	Dry weight
GAE	Gallic acid equivalents
EGCG	Epigallocatechin gallate
EGC	Epigallocatechin
ECG	Epicatechin gallate
EC	Epicatechin
DMSO	Dimethyl sulfoxide
DPPH	2,2-diphenyl-1-picrylhydrazyl
ABTS	2,2′-azino-bis(3-ethylbenzothiazoline-6-sulfonic acid)
