# Supplementary figures and images for: Ku2: A Novel Korean Purple-Green Tea Germplasm (Camellia sinensis) with Enhanced Polyphenols and Antioxidant Activity
Source: Plants (Basel). 2025 Sep 2;14(17):2742. doi: 10.3390/plants14172742 (PMC12430521; doi:10.3390/plants14172742)

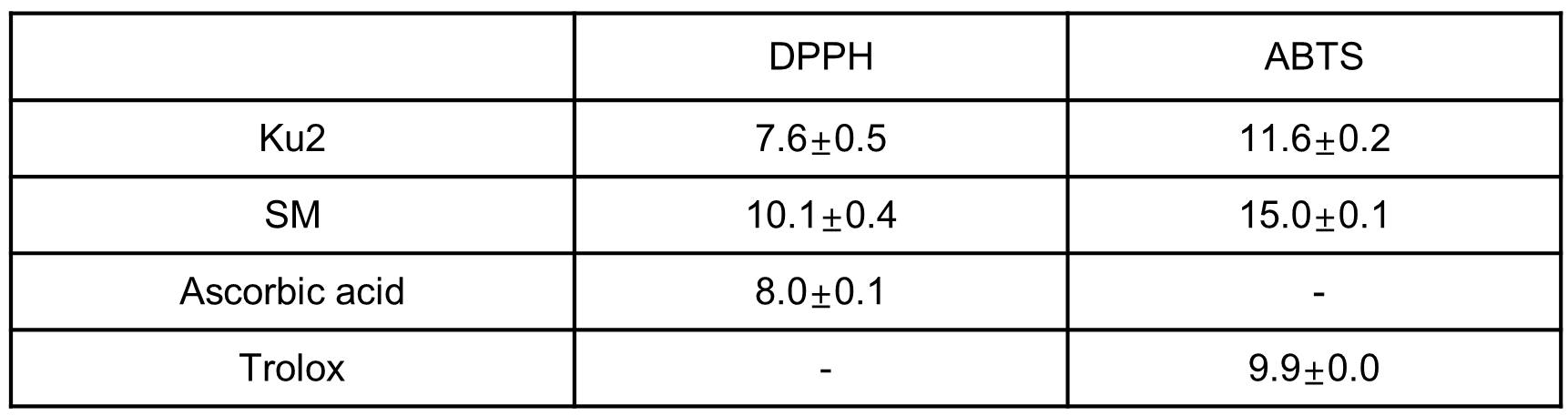

Supplement: Supplementary file 1 [file plants-14-02742-s001.zip › Table S1.TIF]
